# Morphological Parameters in Quadriceps Muscle Were Associated with Clinical Features and Muscle Strength of Women with Rheumatoid Arthritis: A Cross-Sectional Study

**DOI:** 10.3390/diagnostics11112014

**Published:** 2021-10-29

**Authors:** Leonardo Peterson dos Santos, Rafaela Cavalheiro do Espírito Santo, Émerson Pena, Lucas Denardi Dória, Vanessa Hax, Claiton Viegas Brenol, Odirlei André Monticielo, Rafael Mendonça da Silva Chakr, Ricardo Machado Xavier

**Affiliations:** 1 Laboratório de Doenças Autoimunes, Universidade Federal do Rio Grande do Sul (UFRGS), Porto Alegre 90035-903, RS, Brazil; rafaela.esef@gmail.com (R.C.d.E.S.); emersonpena1997@rede.ulbra.br (É.P.); ldoria_pes@hcpa.edu.br (L.D.D.); vanessahax@gmail.com (V.H.); claiton.brenol@gmail.com (C.V.B.); omonticielo@yahoo.com.br (O.A.M.); rafaelchakr@gmail.com (R.M.d.S.C.); rxavier10@gmail.com (R.M.X.); 2 Serviço de Reumatologia, Hospital de Clínicas de Porto Alegre (HCPA), Porto Alegre 90035-903, RS, Brazil; 3 Faculdade de Medicina, Programa de Pós-Graduação em Medicina: Ciências Médicas, Universidade Federal do Rio Grande do Sul (UFRGS), Porto Alegre 90035-903, RS, Brazil

**Keywords:** rheumatoid arthritis, muscle mass, ultrasound, muscle strength, functionality, sarcopenia

## Abstract

Background: Rheumatoid arthritis (RA) is an autoimmune, inflammatory and chronic disease that may lead to loss of muscle mass, muscle strength and decreased functionality. Our objectives are to assess the quadriceps muscle morphology by ultrasound (MU) and verify its associations with clinical features, muscle strength and physical function in RA patients. Methods: In this cross-sectional study, RA women (≥18 years) were included. Morphological parameters in quadriceps muscle consisted of the muscle thickness and pennation angle of rectus femoris (RF), vastus intermedius (VI) and vastus lateralis (VL). RA activity was measured by a 28-joint disease activity score (DAS28), muscle strength by handgrip and chair stand tests, and physical function by health assessment questionnaire (HAQ), timed-up-and-go (TUG) test and short physical performance battery (SPPB). Results: Fifty-five patients were included (age: 56.73 ± 9.46 years; DAS28: 3.08 ± 1.29). Muscle thickness in RF, VI and VL were negatively associated with age (RF, *p* < 0.001; VI, *p* = 0.013; VL, *p* = 0.002) and disease duration (RF, *p* < 0.001; VI, *p* = 0.005; VL, *p* = 0.001), and were positively associated with handgrip strength (RF, *p* = 0.015; VI, *p* = 0.022; VL, *p* = 0.013). In addition, decreased muscle thickness in VI (*p* = 0.035) and a smaller pennation angle in RF (*p* = 0.030) were associated with higher DAS-28 scores. Conclusion: Quadriceps muscle morphology by ultrasound appears to be affected by age, disease duration, disease activity and muscle strength in patients with RA. MU can be a useful method to evaluate the impact of the disease on skeletal muscle.

## 1. Introduction

Rheumatoid arthritis (RA) is an autoimmune, inflammatory and chronic arthropathy [1] that may present extra-articular manifestations [2,3]. Changes in body composition such as reduced fat-free mass, mainly appendicular lean mass, with stable or increased fat mass are examples of extra-articular manifestation [4,5,6,7,8]. Furthermore, changes in body composition are strongly associated with long-term disability and premature mortality [9]. Studies demonstrated that RA patients who receive adequate intensive treatment have ~10% proportionally less appendicular lean mass compared to healthy controls [10,11]. Additionally, RA patients are shown to have lower muscle mass adjusted by fat mass and lower muscle density, a composite index of intramuscular fat substitution, compared to a healthy representative sample [12,13]. Factors such as increased pro-inflammatory cytokines [2,3,14], glucocorticoids use [15], exercise intolerance and sedentary behavior [16] are associated with muscle loss in RA patients.

There are several methods to assess appendicular muscle mass such as magnetic resonance imaging (MRI), computed tomography (CT), dual-energy radiological absorptiometry (DXA) and bioimpedance (BIA) [17,18,19]. MRI and CT are non-invasive assessments, considered the gold standard for assessing muscle mass. However, the high costs of the equipment and the lack of portability make them uncommon tools in clinical practice [20]. DXA is also a non-invasive assessment and it is the most widely available instrument for determining muscle quantity. Nevertheless, DXA measures fat-free mass, not muscle mass. Furthermore, different instrument brands and because they are not portable instruments are additional limitations of the method [18,21,22,23]. BIA also measures muscle mass indirectly. The assessment method estimates muscle mass based on the electrical conductivity of the entire body [18,24]. BIA equipment is affordable, widely available and portable [18]. However, like other methods of assessing muscle mass, BIA also has limitations. The variation in equipment brands, as well as the patient’s hydration status, which makes it difficult to differentiate between muscle mass and internal organs, are disadvantages of the method [18]. So, although the methods have good validity and agreement, their applications in clinical practice, as well as in longitudinal studies, have been limited.

In this sense, the European Working Group on Sarcopenia in Older People 2 (EWGSOP2) suggests new alternatives or tools to assess muscle mass [18]. Muscle ultrasound (MU) is a tool used to assess the thickness and architecture of muscle mass, being relatively fast, safe and portable [25,26]. The MU has recently expanded into clinical practice to aid in the diagnosis of sarcopenia in older adults [18]. The Group of the European Geriatric Medicine Society (EuGMS), in 2018 [27], proposed a consensus protocol for the use of ultrasound involving five components, which MU is able to assess: muscle thickness, cross-sectional area, fascicle length, pennation angle, and echogenicity. This same group, in 2021 [28], carried out an update of the assessment method, presenting its relevance and highlighting the need for standardization in assessments, as well as the need for evolution regarding its guidelines. The MU is reliable and valid to assess muscle thickness in the elderly when compared to other methods such as MRI, CT and DXA [25].In addition, MU evaluation also demonstrated a good intra-class correlation coefficient (ICC) between inter and intra-examiners [25,26].

This method has been used to assess muscle mass in neuromuscular diseases, sarcopenia and inpatients [18,20,29]. In RA patients, there is limited experience with MU to assess muscle thickness and muscle architecture [30]. Matschke et al. (2010) demonstrated that RA patients have a smaller cross-sectional area of the vastus lateralis (−13.9%) and a smaller pennation angle (−12.5%) when compared to healthy patients matched for age and sex [30]. The angle of pennation is defined as the angle between the insertion of the muscle fascicle and the deep aponeurosis [31,32], and is related to the muscle’s capacity to generate force [33]. More recently, Blum et al. (2020) also demonstrated that RA patients showed smaller muscle thickness (−23.3%) and pennation angle (−14.1%), which is the angulations of muscle fibers relative to the line of action of muscle in pennate muscles, compared with healthy women [34].

Therefore, our objectives are (1) to further evaluate muscle thickness and pennation angle of the quadriceps muscle and (2) to verify the associations between the morphological parameters of the quadriceps muscle assessed by MU and clinical features, muscle strength and physical function in female patients with RA.

## 2. Materials and Methods

### 2.1. Study Design

This was a cross-sectional study that involved 55 RA patients. We reported this study according to STROBE Checklist [35].

### 2.2. Settings

The present study was performed at the Rheumatology Division of Hospital de Clínicas de Porto Alegre (HCPA) between May 2018 and August 2021. The institutional review board of the Universidade Federal do Rio Grande do Sul, Hospital de Clínicas de Porto Alegre, Brazil (registered under number 18-0071) approved this study, and the declaration of Helsinki principles were followed. Data collection was composed by clinical features, muscle morphology assessment by MU, muscle strength and physical function.

### 2.3. Participants

Fifty-five female RA patients, aged ≥18 years, followed at the outpatient clinic of a single reference university hospital were consecutively included. RA patients had to fulfill the 2010 American College of Rheumatology (ACR) criteria. Patients were excluded if they had any neuromuscular and metabolic diseases, inability to walk or flex the knee and knee replacement. Additionally, juvenile RA patients were also excluded. All patients gave written consent before joining the study.

### 2.4. Variables

#### 2.4.1. Clinical Features

Age, disease duration (years), race, bone erosions, data about rheumatoid factor (RF), anti-cyclic citrullinated peptides (anti-CCP), C-reactive protein (CRP), and treatment regimen were assessed by a review of medical records.

#### 2.4.2. Disease Activity

Disease activity in RA patients was assessed by the 28-joint Disease Activity Score (DAS28). The DAS28 considers 28 tender and swollen joint counts, general health (GH; patient assessment of disease activity using a 100 mm visual analog scale (VAS) with 0 = best, 100 = worst), plus levels of C-reactive protein (mg/L) [36]. Scores below <2.6 indicated remission, from ≥2.6 to <3.2 low, from ≥3.2 to ≤5.1 moderate, and greater than >5.1 indicated high disease activity [37].

#### 2.4.3. Muscle Ultrasound (MU)

Morphological parameters of the quadriceps muscle were assessed by MU according to standardization recommendations and consisted of muscle thickness and pennation angle measures [28]. The participants were required to lay supine on the examination table, with arms and legs relaxed and in a natural resting position. The images of rectus femoris (RF), vastus intermedius (VI) and vastus lateralis (VL) of anterior portion were taken at 50% of the thigh length from the greater trochanter to the lateral knee joint space [38,39]. Each midpoint was clearly marked on the skin with a surgical pen to ensure proper placement of the probe across repeated scans. Thus, in a neutral tilt, the probe was lightly placed over the pen marking perpendicular to the long axis of the limb to obtain a longitudinal ultrasound image of the site. For each measurement, a generous amount of conductive gel was applied to prevent direct contact between the probe and skin surface. In addition, the images were acquired under the lightest possible compression, avoiding distortion of the muscle tissue. All assessments were performed by a single experienced technician (Santos, LP) using a real-time ultrasound device (Esaote S.p.A MyLab 50 X Vision; São Paulo, Brazil) equipped with a 10–18 MHz linear transducer. B-mode muscle images from the selected sites were captured and used. Whenever required, to ensure a better view of the image, depth adjustment was allowed. After proper visualization, the image was captured (“frozen”) and stored on the device’s hard disk as a JPEG file. The examiner repeated this procedure three times in each of the three sites, resulting in nine saved images from each patient.

**Figure 1:**
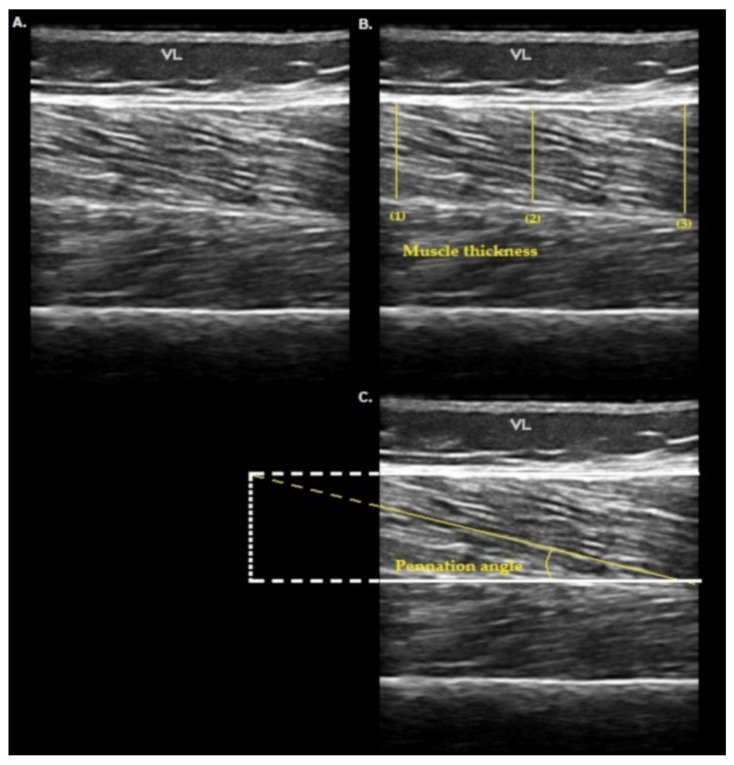
Ultrasound image showing vastus lateralis muscle architecture parameters. (**A**) Vastus lateralis image, (**B**) assessment of muscle thickness and (**C**) assessment of pennation angle. VL: vastus lateralis; (1): first parallel line drawn at right angles between the superficial and deep aponeurosis along ultrasonography image for muscle thickness; (2): second parallel line drawn at right angles between the superficial and deep aponeurosis along ultrasonography image for muscle thickness; (3): Third parallel line drawn at right angles between the superficial and deep aponeurosis along ultrasonography image for muscle thickness.

The collected images were downloaded for further analysis with the ImageJ program version 1.52a (National Institutes of Health, Maryland, USA). However, prior to image selection, two evaluators (Santos, LP and Pena, E) together screened the image bank to identify and discard images associated with technical issues, such as excessive compression or probe tilting. Thereafter, a single image was selected from each evaluated site (therefore, three images per patient) [40]. Among the images available at the same location, preference was given to those with darker muscle tones as visual criteria that suggest lesser compression of the probe [40] and better visualization of the fascicle. If all images collected for a given location did not meet established technical standards that location was considered “absent”, and the patient was excluded from the analysis. For the assessment of the muscle thickness, the distance between deep and superficial aponeuroses was considered, and it was calculated through the mean value of three parallel lines drawn at right angles between the superficial and deep aponeuroses along each ultrasonography image [34]. In addition, the fascicle with the best visualization of each image was used for pennation angle analysis. Pennation angle was calculated as the angle between the muscle fascicle and the deep aponeurosis [34] (Figure 1A–C).

#### 2.4.4. Muscle Strength Assessment

Muscle strength was assessed by the handgrip strength test and chair stand (5-repetition) test. Handgrip strength was measured using a handheld dynamometer (Jamar Hydraulic Hand Dynamometer, Preston, CT, USA). The patient was instructed to squeeze the handle as hard as possible for 5 s, and the maximal isometric voluntary contraction (MIVC) was quantified. The measurement was repeated after a recovery period of 60 s, and the highest value of three MIVCs was considered for analysis. Handgrip strength values <16 kg (women) was considered indicative of muscle weakness [18,41]. For patients in whom joint involvement impaired testing, adjustments were made to the handheld dynamometer as described elsewhere [42].

The chair stand (5-repetition) test was performed as a measure of lower limb muscle strength. This is a timed test in which the participant is asked to get up from a chair and sit down five times as quickly as possible, without using his or her arms. The amount of time to perform the five repetitions is used as a measure. The performance >15 s for five rises was considered low muscle strength [18,43].

#### 2.4.5. Physical Function

Physical function was assessed by the Health Assessment Questionnaire (HAQ), Timed-up-and-go (TUG) test and Short Physical Performance Battery (SPPB). The HAQ is a questionnaire about the level of difficulty in performing activities of daily living [44]. The HAQ classification is given as mild (HAQ 0–1), moderate (HAQ 1–2) and severe impairment (HAQ > 2) [45]. The TUG test measures the time for an individual to rise from a chair, walk 3 m to a marker (cone), turn 180°, return to the chair and sit down. Time was recorded by a stopwatch, and participants were instructed not to use their hands when rising from or sitting back down on the chair. Any time ≥20 s was considered slow (low speed) [46]. The SPPB, in turn, is a widely used and simple test to measure lower extremity function through observed completion of tasks that mimic daily actions. The SPPB includes three physical performance domains: balance, walking speed and five-time sit-to-stand test. SPPB score ranges from 0 through 12. A score ≤8 points indicates poor physical performance [42,47,48].

### 2.5. Statistical Analysis

The Shapiro–Wilk method was used to test for normality. Results are expressed as mean ± standard deviation (SD), median (interquartile range), and number (%), as appropriate. Cross-sectional analyzes T-tests for independent samples were performed to compare groups as appropriate. Pearson’s or Spearman’s correlation coefficients were explored to assess the associations among muscle mass (muscle thickness and pennation angle) with clinical features, muscle strength and physical function. Correlations were ranked as suggested by Dancey and Reidy [49]: r = 1.0 indicates perfect association; r = 0.7–0.9, strong association; r = 0.4–0.6, moderate association; r = 0.1–0.3, weak association; and r = 0, no association. Multiple linear regression analyses were performed to investigate the associations of the muscles thickness (RF, VI and VL) with age, disease duration and handgrip strength. These three variables were chosen because both are correlated with the same musculature in Pearson’s or Spearman’s analysis. Still, in multiple linear regression analyzes, multicollinearity was verified by the variance inflation factor (VIF). The acceptable VIF in our study was <2. The significance level was set at *p* ≤ 0.05 for all analyzes. Statistical analyzes were performed in Statistical Package for Social Sciences (SPSS) 17.0.

## 3. Results

Fifty-five RA women were included in the study. The mean age was 56.73 ± 9.46 years and the mean body mass index (BMI) was 28.31 ± 5.01 kg/m^2^. The median disease duration was 10.00 (5.00–19.00) years, and the mean of DAS28-CRP was 3.08 ± 1.29. Regarding treatment, 36 patients (65.5%) were under methotrexate (MTX), 12 patients were using conventional synthetic disease-modifying antirheumatic drugs (csDMARDs) without MTX (21.8%), four patients (7.2%) were using only glucocorticoids without any other medication and three patients (5.5%) were not using any medication. Demographic and clinical features data are described in Table 1.

Regarding muscle strength, 33 patients (60%) showed low strength (<16 kg) according to the EWGSOP2 cut-off points. For the chair stand (5-repetition) test, we obtained data from 32 patients, and 13 (40.6%) showed low strength (>15 s). The HAQ was performed by 39 patients, and 24 (61.5%) had mild, 13 (33.3%) moderate and two (5.1%) severe impairment. Due to the small number of patients categorized as severe impairment by the HAQ, we grouped patients with moderate and severe impairment (>1 score). The SPPB test was performed by 32 patients, and 10 (31.25%) showed low performance (≤8 point score) according to the EWGSOP2 cut-off. Forty-two patients performed the TUG test as a measure of the objective function. Only one patient (2.4%) presented low performance (>20 s) according to the EWGSOP2 cut-off. Data on muscle thickness and pennation angle of the quadriceps muscles, as well as data on muscle strength and physical function of RA patients, are shown in Table 2.

Regarding DAS28-CRP, 25 patients were in remission (score < 2.6) and 30 patients were in non-remission (score ≥ 2.6). The mean disease activity of patients in remission was 1.99 ± 0.35 and non-remission was 4.00 ± 1.04. No statistically significant differences were found between the mean on muscle thicknesses (RF, *p* = 0.890; VI, *p* = 0.218; VL, *p* = 0.159) and pennation angles (RF, *p* = 0.321; VI, *p* = 0.278; VL, *p* = 0.390) of women in remission when compared to women in non-remission.

**Figure 2:**
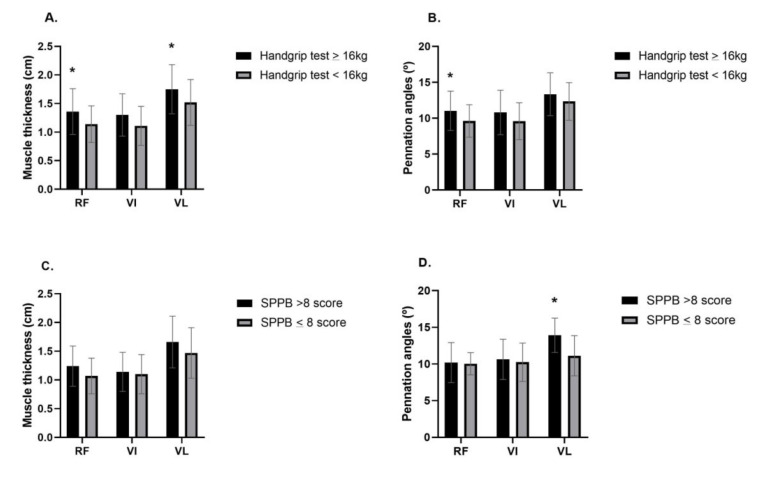
(**A**,**B**) Muscle thickness and pennation angle of patients according to the level of muscle strength assessed by handgrip test. (**C**,**D**) Muscle thickness and pennation angle of patients according to the level of physical performance assessed by SPPB. RF: rectus femoris; VI: vastus intermedius; VL: vastus lateralis; cm: centimeters; °: degrees; kg: kilograms; SPPB: Short Physical Performance Battery; *: statistically significant difference (*p* ≤ 0.05). Data presented as mean ± SD.

**Figure 3:**
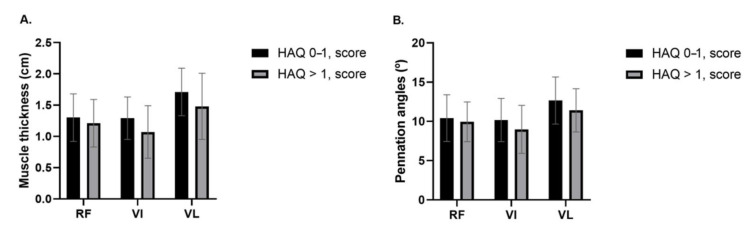
(**A**) Muscle thickness of patients according to the level of physical function assessed by HAQ. (**B**) Pennation angle of patients with low physical function assessed by HAQ. RF: rectus femoris; VI: vastus intermedius; VL: vastus lateralis; cm: centimeters; °: degrees; HAQ: Health Assessment Questionnaire. Data presented as mean ± SD.

Patients with low muscle strength by handgrip test (<16 kg) showed smaller muscle thicknesses of RF (*p* = 0.030) and VL (*p* = 0.046) (Figure 2A), as well as a smaller pennation angle of RF (*p* = 0.042) than women with muscle strength (≥16 kg) (Figure 2B). The muscle thickness of VI (*p* = 0.055), and pennation angles of VI (*p* = 0.122) and VL (*p* = 0.193) showed no statistically significant differences. In addition, in patients with low performance by SPPB (≤8 score), no statistically significant differences were found in muscle thickness (RF, *p* = 0.210; VI, *p* = 0.803; VL, *p* = 0.271) (Figure 2C) and pennation angle of RF (*p* = 0.866) and VI (*p* = 0.720). However, these women showed smaller VL pennation angle (*p* = 0.006) than RA women with >8 score (Figure 2D). Regarding to chair stand (5-repetition), no statistically significant differences were found in muscle thickness (RF, *p* = 0.196; VI, *p* = 0.447; VL, *p* = 0.265) and pennation angles (RF, *p* = 0.549; VI, *p* = 0.431; VL, *p* = 0.253). For the HAQ assessment, due to the small number of patients categorized as severe impairment, we grouped the patients in moderate and severe impairment (>1 score). No statistically significant differences were found in muscle thickness (RF, *p* = 0.443; VI, *p* = 0.084; VL, *p* = 0.115) (Figure 3A) and pennation angle (RF, *p* = 0.613; VI, *p* = 0.216; VL, *p* = 0.198) (Figure 3B).

Lastly, it was not possible to perform analysis between the TUG test extracting patients with low physical performance due to the small number of participants.

Finally, among RA women who were or were not using glucocorticoids, there was no statistically significant difference in the analysis of muscle thickness (RF, *p* = 0.248; VI, *p* = 0.094; VL, *p* = 0.807), pennation angles (RF, *p* = 0.078; VI, *p* = 0.066; VL, *p* = 0.985), handgrip strength (*p* = 0.774), chair stand (*p* = 0.472), HAQ (*p* = 0.850), TUG test (*p* = 0.209) and SPPB (*p* = 0.746).

### Relationship among Morphological Parameters in the Quadriceps Muscle Assessed by MU and Clinical Features, Muscle Strength and Physical Function

**Figure 4:**
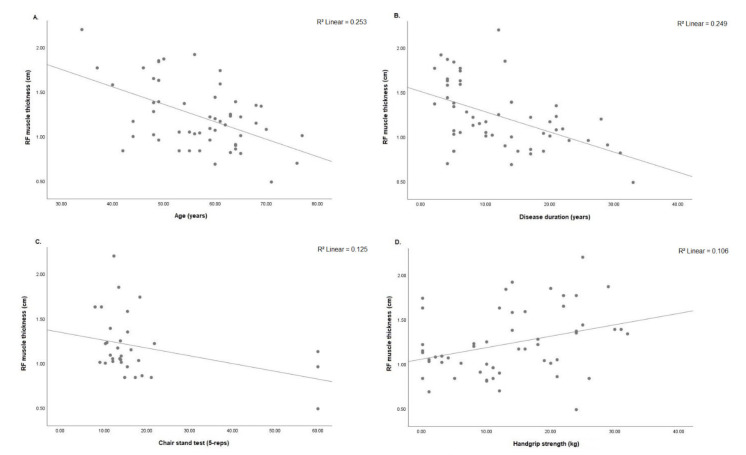
(**A**) Correlation analysis was significant between RF muscle thickness and age (*n* = 55, r = −0.503; *p* < 0.001), (**B**) disease duration (*n* = 55, r = −0.493; *p* < 0.001), (**C**) chair stand test (*n* = 32, r = −0.368; *p* = 0.038) and (**D**) handgrip strength (*n* = 55, r = 0.325; *p* = 0.015). RF: rectus femoris; cm: centimeters; 5-reps: five repetitions; kg: kilograms.

**Figure 5:**
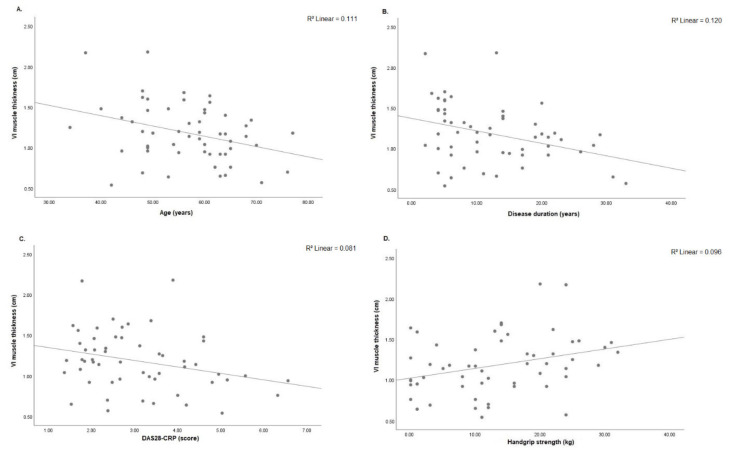
(**A**) Correlation analysis was significant between VI muscle thickness and age (*n* = 55, r = −0.333; *p* = 0.013), (**B**) disease duration (*n* = 55, r = −0.372; *p* = 0.005), (**C**) DAS28-CRP (*n* = 55, r = −0.285; *p* = 0.035) and (**D**) handgrip strength (*n* = 55, r = 0.309; *p* = 0.022). VI: vastus intermedius; DAS28-CRP: 28-joint disease activity score, C-reactive protein; kg: kilogram.

**Figure 6:**
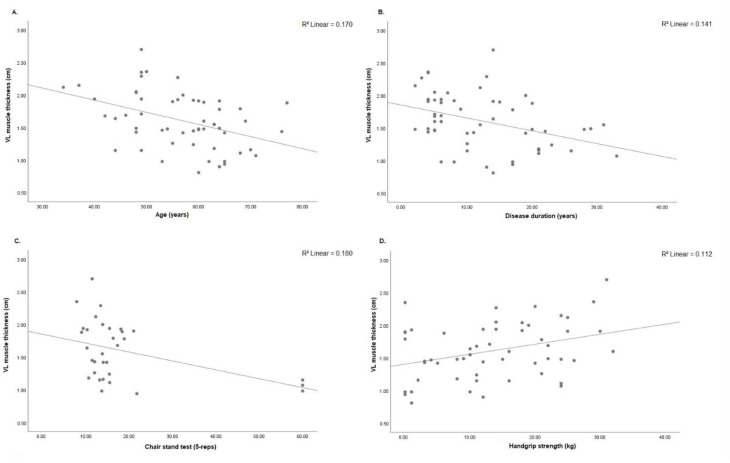
(**A**) Correlation analysis was significant between VL muscle thickness and age (*n* = 55, r = −0.412; *p* = 0.002), (**B**) disease duration (*n* = 55, r = −0.426; *p* = 0.001), (**C**) chair stand test (*n* = 32, r = −0.394; *p* = 0.026) and (**D**) handgrip strength (*n* = 55, r = 0.335; *p* = 0.013). VL: vastus lateralis; cm: centimeters; 5-reps: five repetitions; kg: kilograms.

**Figure 7:**
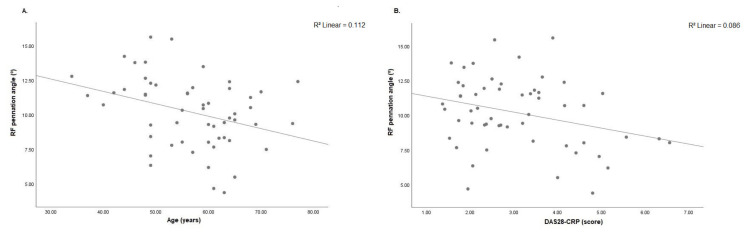
(**A**) Correlation analysis was significant between RF pennation angle and age (*n* = 55, r = −0.334; *p* = 0.013) and (**B**) DAS28-CRP (*n* = 55, r = −0.293; *p* = 0.030). RF: rectus femoris; °: degrees; DAS28-CRP: 28-joint disease activity score, C-reactive protein.

Decreased RF muscle thickness was associated with older age (r = −0.503; *p* < 0.001), longer disease duration (r = −0.493; *p* < 0.001) and prolonged chair stand test (r = −0.368; *p* = 0.038). On the other hand, RF muscle thickness was positively associated with handgrip strength (r = 0.325; *p* = 0.015) (Figure 4A–D). VI muscle thickness was negatively associated with age (r = −0.333; *p* = 0.013), disease duration (r = −0.372; *p* = 0.005) and DAS28-CRP (r = −0.285; *p* = 0.035). On other hand, better VI muscle thickness was associated with handgrip strength (r = 0.309; *p* = 0.022) (Figure 5A–D).VL muscle thickness was negatively associated with age (r = −0.412; *p* = 0.002), disease duration (r = −0.426; *p* = 0.001) and chair stand test (r = −0.394; *p* = 0.026). On other hand, VL muscle thickness was associated with handgrip strength (r = 0.335; *p* = 0.013) (Figure 6A–D). RF pennation angles was negatively associated with age (r = −0.334; *p* = 0.013) and DAS28-CRP (r = −0.293; *p* = 0.030) (Figure 7A,B).

Lastly, age explained 33% of the RF muscle thickness decrease, 19% of the VI muscle thickness decrease and 27% of the VL muscle thickness decrease. Disease duration explained 33% of the decrease in RF muscle thickness, 23% of the decrease in VI muscle thickness, and 23% of the decrease in VL muscle thickness. On the other hand, handgrip strength explained 27% of the increase in the RF muscle thickness, 23% of the increase in the VI muscle thickness and 25% of the increase in the VL muscle thickness. More details are described in Table 3.

## 4. Discussion

The main findings of this study were that RA patients with lower muscle strength present impairment of muscle thickness of rectus femoris (RF) and vastus lateralis (VL), as well as RF pennation angle. In addition, the VL pennation angle also appears worse when patients have lower SPPB. In addition, the muscle thickness of RF and VL were associated with age, disease duration, chair stand (5-repetition) and handgrip strength, while the VI was associated with age, disease duration, DAS28-CRP and handgrip strength. RF pennation angles were only associated with age and DAS28-CRP.

There are limited data on muscle morphology assessment by ultrasound in RA patients. Blum et al. (2020) [34] recently found mean muscle thickness and pennation angle in patients with RA of 1.37 ± 0.34 cm and 8.65 ± 2.36°, respectively. Our findings of mean VL muscle thickness and VL pennation angle were higher. However, RA patients of the Blum et al. (2020) study [34] had moderate mean disease activity, while our RA patients had low disease activity. In fact, our VL muscle thickness findings (1.61 ± 0.43 cm) were lower than those from the healthy group in this same study [34]. Santo et al. (2020) [50] demonstrated that RA patients in low disease activity had a higher appendicular lean mass index (ALMI) than RA patients in moderate/high disease activity. Thus, disease activity may affect muscle mass and could explain the greater muscle thickness and pennation angle found in our study compared to Blum et al. (2020) [34].

Additionally, Matschke et al. (2010) [30] evaluated patients with stable RA and demonstrated a mean of pennation angle lower than our patients. However, RA patients of Matschke et al. (2010) [30] were using higher daily doses of glucocorticoids (mean dose 7.3 mg) compared to our RA patients (mean dose 5.0 mg). The same occurs with patients of the Blum et al. (2020) study [34] (mean dose 10.9 mg). Yamada et al. (2020) [15] demonstrated that the use of glucocorticoids directly affects muscle mass. It may explain the greater pennation angle found in our study compared to Matschke et al. (2010) [30] and Blum et al. (2020) [34] since our patients were using lower daily doses of glucocorticoids.

Muscle thickness and pennation angles were weakly or moderately associated with age, disease duration, disease activity and muscle strength in our sample. Only Blum et al. (2020) [34] evaluated associations of muscle thickness and pennation angle with clinical features and physical function in RA patients and there was no association among muscle thickness and pennation angle with age, disease duration, disease activity, physical function by HAQ, isokinetic knee muscle strength, pain and glucocorticoids [34]. We believe that our associations were found due to the larger sample size compared to Blum et al. (2020) [34]. It is known that older RA patients have less muscle mass compared to younger patients [51]. In addition, patients with RA, even at an early stage, are already affected with loss of lean mass and gain in fat mass [52,53]. Our study demonstrated that RA patients with lower muscle mass have lower muscle strength and lower physical performance. Furthermore, the association of muscle thickness and pennation angle with disease activity (DAS28-PCR) indicates that the inflammatory burden may also be an important factor in regard to the morphological impairment of the quadriceps muscle in women with RA. Lastly, we found a low ability to explain the variability of the results performed in multivariate analyzes between age, disease duration and handgrip strength. In this sense, the interpretations of these results must be cautious. Although the correlations are low to moderate and the explanatory power between the variables age, disease duration and handgrip strength is also low, it should be noted that this is a pilot study with a limited sample. Nevertheless, data on muscle ultrasound assessment in the literature are limited, especially in rheumatoid arthritis. Thus, we believe that larger samples could better explain the results of muscle strength, physical function and clinical characteristics of these patients.

Despite these, our study is relevant considering that the ultrasound technique can assess muscle quality and quantity, being associated with age, disease duration, disease activity and muscle strength in RA. In addition, our findings of muscle thickness in RA women were lower than those of the healthy age-matched women found in the literature [34]. Therefore, muscle ultrasound seems to be a promising tool that may be more easily used in clinical practice to assess muscle morphology in RA patients.

This cross-sectional study has some limitations. The first limitation was the absence of a control group with healthy individuals matched by age and gender. A second limitation was the absence of data of muscle mass quantity evaluated by a gold-standard method. In the future, this data may be used to validate ultrasound for the assessment of muscle mass in RA patients. Furthermore, any causal inference should not be done due to the study’s cross-sectional design. Lastly, the external validity of our results is limited to RA women.

## 5. Conclusions

In conclusion, the impairment of muscle thickness and pennation angle assessed by ultrasound appears to be influenced by age, disease duration, disease activity and muscle strength in women with rheumatoid arthritis. Thus, controlling disease activity, in addition to improving the clinical aspects of patients, may directly influence muscle morphology. Finally, the muscle ultrasound variables may provide important information for clinical follow-up of RA patients. Joint US is a technique that has been increasingly used by rheumatologists in their offices during routine care of RA patients. Thus, the possibility of evaluating muscle status quickly and accurately would be of great usefulness in the screening for sarcopenia, with a good chance of adoption in clinical practice. However, it is necessary to analyze a larger number of patients, including men, to confirm these findings and to correlate with functional outcomes.

## Figures and Tables

**Table 1:** Demographic and clinical features of the study sample.

Demographic	RA Patients (*n* = 55)
Age (years), mean ± SD	56.73 ± 9.46
Weight (kg), mean ± SD	70.34 ± 13.46
BMI (kg/m^2^), mean ± SD	28.31 ± 5.01
Disease duration (years), median (IQR)	10.00 (5.00–19.00)
Race, (*n* [%])	
Caucasian	43 [78.2]
Other	12 [21.8]
Positive rheumatoid factor, (*n* [%])	49 [89.1]
Anti-CCP, (*n* [%])	
Positive	33/38 tested [86.8]
Erosions, (*n* [%])	42 [76.4]
Disease activity (DAS-28—CRP)	
DAS-28—CRP, mean ± SD	3.08 ± 1.29
Remission (<2.6), (*n* [%])	25 [45.5]
Low (2.6 to <3.2), (*n* [%])	7 [12.7]
Moderate (3.2 < 5.1), (*n* [%])	19 [34.5]
High (>5.1), (*n* [%])	4 [7.3]
CRP (L/mg), median (IQR)	6.70 (3.00–13.90)
Treatment regimen	
MTX (*n* [%])	
MTX in monotherapy	27 [49.1]
MTX with concurrent csDMARDs	9 [16.4]
Do not use	19 [34.5]
MTX dose (mg/week), median (IQR)	18.75 (15.00–20.00)
csDMARDs without MTX, (*n* [%])	12 [21.8]
bDMARDswith concurrent MTX, (*n* [%])	6 [10.9]
bDMARDs with concurrent csDMARDs, (*n* [%])	3 [5.5]
Glucocorticoids, (*n* [%])	19 [34.5]
Glucocorticoids (mg/day), median (IQR)	5.00 (5.00–10.00)

RA: rheumatoid arthritis; *n*: number; SD: standard deviation; kg: kilogram; kg/m^2^: kilogram per square meter; IQR: interquartile range; %: percent; Anti-CCP: anti-cyclic citrullinated peptides; DAS28: Disease Activity Score 28; CRP:C-reactive protein; mg/L: milligram per liter; MTX: methotrexate; csDMARDs: conventional synthetic disease-modifying antirheumatic drugs (in this case, leflunomide and sulfasalazine); bDMARDs: biologic disease-modifying antirheumatic drugs (in this case, adalimumab, etanercept and abatacept); mg/week: milligram per week; mg/day: milligram per day.

**Table 2:** Muscle thickness, pennation angle, muscle strength and physical function outcomes data of RA patients.

Outcomes	*n*	
Muscle thickness (cm), mean ± SD		
Rectus femoris	55	1.23 ± 0.37
Vastus intermedius	55	1.18 ± 0.36
Vastus lateralis	55	1.61 ± 0.43
Pennation angle (°), mean ± SD		
Rectus femoris	55	10.18 ± 2.54
Vastus intermedius	55	10.07 ± 2.82
Vastus lateralis	55	12.74 ± 2.78
Handgrip (kg), mean ± SD	55	13.40 ± 9.37
<16 kg	33	6.97 ± 5.26
≥16 kg	22	23.05 ± 4.59
Chair stand, median (IQR), sec.	32	13.92 (11.59–17.88)
>15 sec.	13	18.36 (15.89–40.88)
<15 sec.	19	12.03 (10.30–13.80)
HAQ (score), mean ± SD	39	0.94 ±0.70
0–1 score	24	0.49± 0.36
>1 score	15	1.64 ± 0.49
SPPB (score), mean ± SD	32	9.44 ± 1.83
<8 score	10	7.30 ± 0.82
>8 score	22	10.41 ± 1.22
TUG, median (IQR), sec.	42	8.58 (7.60–9.95)

RA: rheumatoid arthritis; *n*: number; SD: standard deviation; cm: centimeters; °: degrees; kg: kilogram; IQR: interquartile range; sec.: seconds; HAQ: Health Assessment Questionnaire; SPPB: Short Physical Performance Battery; TUG: timed-up-and-go.

**Table 3:** Linear regression models for muscle mass (RF, VI and VL).

Model	Beta	*F*	*R* ^2^	*p*-Value
RF muscle thickness		11.262°	0.398	<0.001
Age (years)	−0.327			0.009
Disease duration (years)	−0.332			0.008
Handgrip strength (kg)	0.211			0.064
VI muscle thickness		4.735°	0.218	0.005
Age (years)	−0.194			0.162
Disease duration (years)	−0.231			0.096
Handgrip strength (kg)	0.236			0.068
VL muscle thickness		6.604°	0.280	0.001
Age (years)	−0.273			0.043
Disease duration (years)	−0.226			0.090
Handgrip strength (kg)	0.247			0.047

RF: rectus femoris; VI: vastus intermedius; VL: vastus lateralis; kg: kilogram.

## Data Availability

Not applicable.
